# Successful Treatment of Bronchial Fistula after Pulmonary Lobectomy by Endobronchial Embolization Using an Endobronchial Watanabe Spigot

**DOI:** 10.1155/2015/425694

**Published:** 2015-04-15

**Authors:** Yuichiro Machida, Makoto Tanaka, Nozomu Motono, Sumiko Maeda, Katsuo Usuda, Motoyasu Sagawa

**Affiliations:** Department of Thoracic Surgery, Kanazawa Medical University, 1-1 Daigaku, Uchinada-machi, Kahokugun, Ishikawa 920-0293, Japan

## Abstract

A bronchial fistula is one of the most serious complications that can occur following pulmonary lobectomy. We herein report a case of bronchial fistula that was successfully treated by endobronchial embolization using an Endobronchial Watanabe Spigot (EWS). A 72-year-old male underwent right lower lobectomy of the lung with nodal dissection for a pulmonary squamous cell carcinoma. A bronchial fistula developed 53 days after surgery. Tube drainage was performed, and air leakage was apparent. Under endoscopic observation, intrathoracic injection of indigo carmine revealed that a fistula existed at the peripheral site of the B^2^ai bronchus. After one EWS (small) was inserted into the B^2^a bronchus tightly using a bronchoscope, the air leakage was stopped. Pleurodesis was further carried out, the thoracostomy tube was subsequently removed, and the patient was discharged. Endobronchial embolization using an EWS is an option for the treatment of a bronchial fistula after pulmonary resection.

## 1. Introduction

After surgery for lung cancer, several complications are observed, such as arrhythmia, pneumonia, and atelectasis [1]. A bronchial fistula is one of the most serious complications, and the treatment is sometimes difficult. We herein report a case of bronchial fistula that was successfully treated by endobronchial embolization using an Endobronchial Watanabe Spigot (EWS).

## 2. Case Presentation

**Figure 1:**
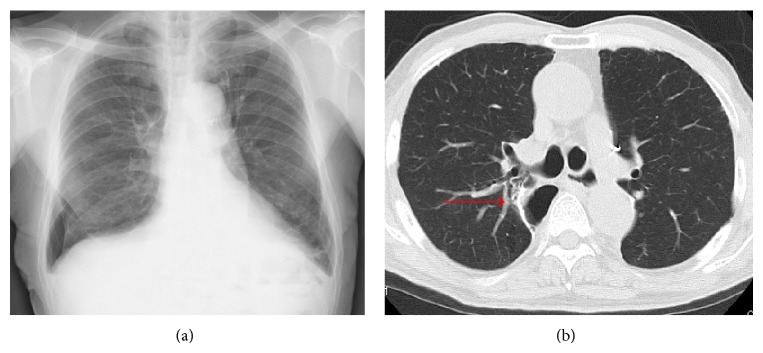
(a) A right pneumothorax is observed in the lower right lung field. (b) The thoracic CT findings suggest that the fistula might exist in the periphery of the B^2^a bronchus (arrow).

**Figure 2:**
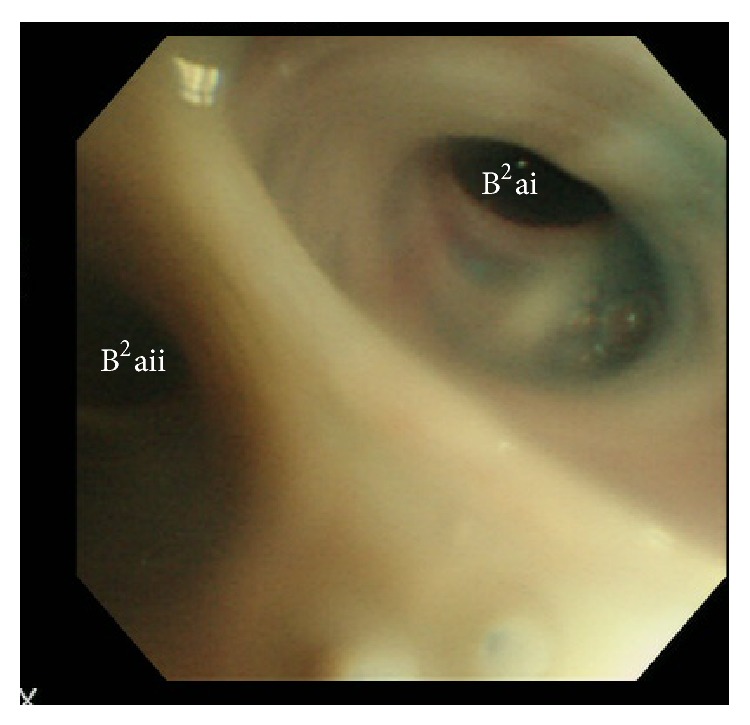
Indigo carmine is running out from the right B^2^ai bronchus.

**Figure 3:**
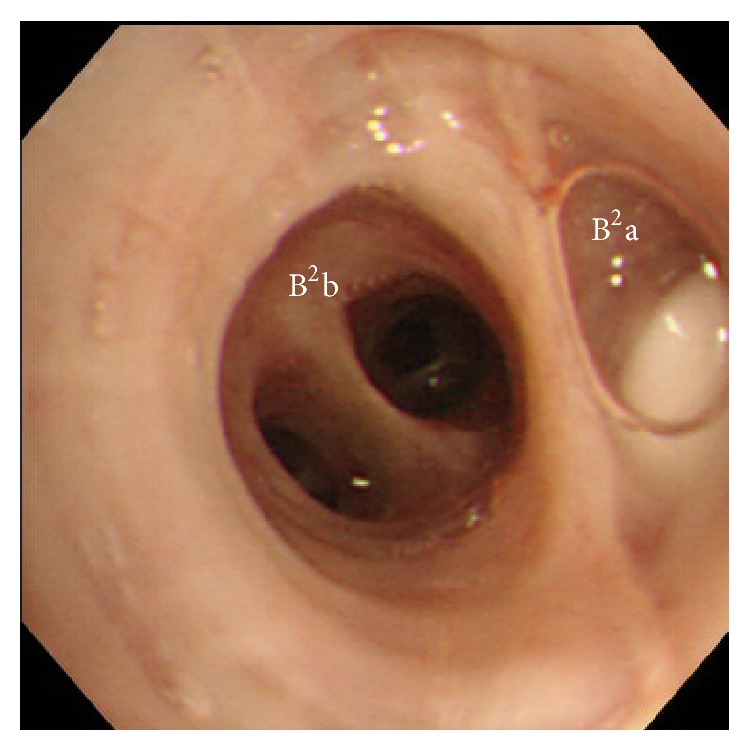
An EWS (small) is tightly inserted into the right B^2^a bronchus.

A 72-year-old male underwent right lower lobectomy of the lung with nodal dissection for pulmonary squamous cell carcinoma. An incomplete interlobar fissure between the upper and lower lobes was present, and these lobes were dissected using staplers. The postoperative course was uneventful and he was discharged from the hospital. However, a right pneumothorax developed 53 days after surgery (Figure 1(a)), and the patient was admitted to our hospital. When a drainage tube was inserted into his thoracic cavity, air leakage was observed. The thoracic computed tomography (CT) findings suggested that the fistula existed in the S^2^a subsegment (Figure 1(b)). Bronchoscopy was carried out in order to identify the responsible bronchus. When indigo carmine was injected via the thoracostomy tube, blue fluid ran from the periphery of the B^2^ai bronchus (Figure 2). Accordingly, one EWS (small) was inserted tightly into the B^2^a (Figure 3), and the air leakage was eliminated. Subsequently, pleurodesis was carried out, and the thoracostomy tube was removed. Complications due to EWS insertion were not observed, and the patient was discharged from the hospital 20 days after the bronchial embolization.

## 3. Discussion

A bronchial fistula is known as one of the most serious complications following thoracic surgery, and the treatment is sometimes difficult due to the poor general and pulmonary conditions of affected patients. Bronchial fistulae sometimes cause empyema, and such patients require a longer hospital stay. There are two types of bronchial fistulae that develop after pulmonary resection: a fistula of the lobar/main bronchial stump or a fistula of a smaller bronchus located at a more peripheral area, like our present case, which was resected due to an incomplete interlobar fissure or the interlobar invasion of cancer. A fistula of the bronchial stump is very serious, but the management of a fistula of a smaller bronchus is still difficult.

Kozower et al. investigated 18,800 resected lung cancer cases in 111 institutions included in the Society of Thoracic Surgeons General Thoracic Database (STSGTD) and determined that air leakage of seven days or more following surgery was observed in 722 cases (8%) and that a bronchial stump fistula was observed in 27 cases (0.3%) [1]. Thoracic surgeons frequently encounter complications associated with air leakage following lung cancer surgery.

**Figure 4:**
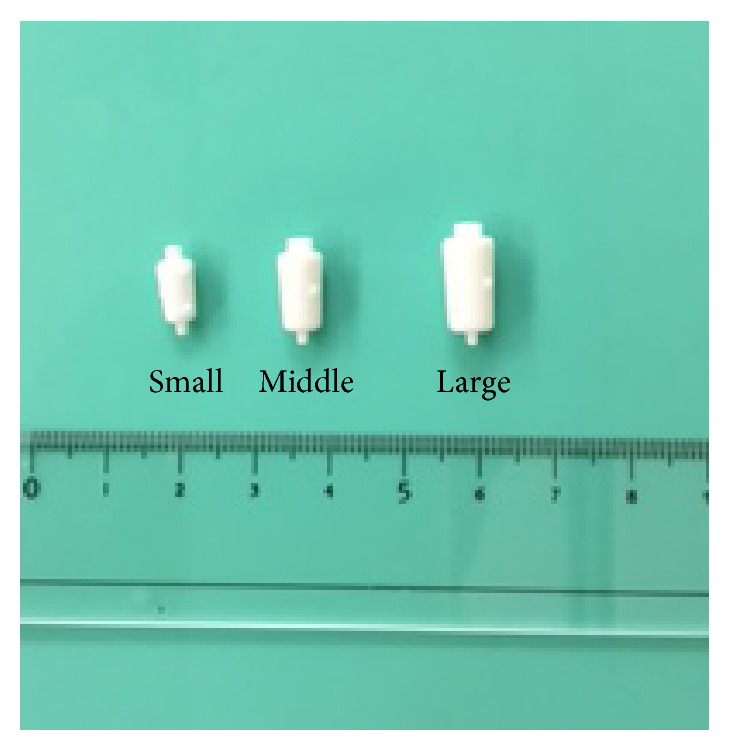
Three types of EWS.

A variety of materials have been used for bronchial embolization. The first case of endobronchial embolization was reported by Rafinski, in which gauze and a polyvinyl sponge were filled under a rigid endoscope [2]. Subsequently, endobronchial embolization using fibrin glue [3] was reported, but the results were inconsistent, probably because the glue was an absorbent material. The EWS used in the present study was made of silicone and specially constructed for endobronchial embolization by Watanabe et al. (Figure 4), and its usefulness for pulmonary air leakage has been reported previously [4].

In this study, indigo carmine was used to identify the responsible bronchus. The indigo carmine injected into the thoracic cavity ran from the periphery of the B^2^ai bronchus, and the patient was diagnosed to have a fistula of the peripheral bronchus at S^2^ai. Since the B^2^ai bronchus was too narrow, an EWS (small) was tightly inserted into the B^2^a bronchus, and the air leakage was eliminated. According to a report by Sasada et al., air leakage was eliminated in 50% of all cases by EWS insertion [5]. An EWS was also reported to be useful in cases of tuberculous empyema with fistula [6] and repeated hemoptysis [7], as long as the responsible bronchus was identified.

Migration is the most common complication associated with endobronchial embolization using an EWS [5]. In our case, even two years after the EWS insertion, no migration of the EWS has been observed. Complications such as a pneumonia or atelectasis have also not been observed. Since there have so far been no reports regarding a long-term follow-up after EWS insertion, further studies are required to confirm the long-term safety of the placement of an EWS, with special attention focused on the potential complications.

Endobronchial embolization using an EWS is a promising option for the treatment of a bronchial fistula after pulmonary lobectomy.
